# Prognostic factors for death after surgery for small intestinal neuroendocrine tumours

**DOI:** 10.1002/bjs5.76

**Published:** 2018-05-28

**Authors:** J. Eriksson, J. E. H. Garmo, C. Ihre‐Lundgren, P. Hellman

**Affiliations:** ^1^ Department of Surgical Sciences Uppsala University Uppsala Sweden; ^2^ Regional Oncological Centre Uppsala University Uppsala Sweden; ^3^ Department of Molecular Medicine and Surgery Karolinska Institutet Stockholm Sweden; ^4^ Cancer Epidemiology Unit, Division of Cancer Studies King's College London London UK

## Abstract

**Background:**

Neuroendocrine tumours of the small intestine (SI‐NETs) are rare gastrointestinal neoplasms with an annual incidence of about one per 100 000. Patients with apparently similar tumours have variable outcomes. The aim of this study was to identify postoperative prognostic factors identifiable after initial surgery.

**Methods:**

This was a nested case–control study of patients with SI‐NETs who were treated between 1961 and 2001. Data were retrieved from the Swedish Cancer Registry. Patients who died from the SI‐NET and corresponding controls (who outlived cases by at least 1 month), matched by age at diagnosis and calendar period, were included. Sex, postoperative symptoms, postoperative **5**‐hydroxyindoleacetic acid (5‐HIAA) values, European Neuroendocrine Tumor Society (ENETS) stage, insufficiency of the tricuspid valve, radical secondary surgery and secondary malignancy were studied as potential prognostic factors.

**Results:**

In total, 1122 patients were included (561 cases, 561 controls). Postoperative factors of prognostic importance included hormone‐related symptoms, stage IV disease, raised levels of 5‐HIAA, insufficiency of the tricuspid valve, secondary surgery not being macroscopically radical and a second malignancy.

**Conclusion:**

Stage and symptomatic disease are important prognostic factors in SI‐NET.

## Introduction

Neuroendocrine tumours originating in the small intestine (SI‐NETs) are uncommon neoplasms with an annual incidence of about one per 100 000^1^. The incidence of SI‐NETs has been increasing in recent decades^2^, ^3^, ^4^. SI‐NETs arise from the enterochromaffin cells of the small bowel, usually in the ileum, and are the most common small bowel malignancy, followed by adenocarcinoma^2^ ^5^. They are malignant tumours with often indolent behaviour. The 5‐year overall survival rate is approximately 70 per cent, and median overall survival is 7–9 years^2^ ^4^, ^6^, ^7^, ^8^, ^9^. Metastasis is common by the time of presentation, making curative treatment difficult. Prognostication has proven a challenge, as patients with seemingly similar tumours have remarkably different outcomes^10^. Previous work^8^ ^10^, ^11^, ^12^, ^13^, ^14^, ^15^ has identified hormonal symptoms, stage IV disease and the indication for primary surgery (with a worse outcome for elective compared with emergency procedures) as important prognostic factors.

The tumours secrete bioactive peptides that are metabolized in the liver. Symptoms are usually evident only once liver metastasis has occurred, unless the patient has a substantial disease burden that can evade hepatic metabolism^5^. These peptides also stimulate collagen production, resulting in a local peritumoral fibrotic reaction, vascular occlusion and insufficiency of the tricuspid valve, causing carcinoid heart disease^15^ ^16^, recognized as one of the strongest predictors of reduced survival^5^ ^17^, ^18^. It has been suggested^19^, ^20^, ^21^ that patients with SI‐NETs have an increased risk of developing second malignancies due to a tumour‐promoting effect of the circulating peptides, as well as a mutagenic effect caused by medical treatment.

Surgery is often considered beneficial, even in patients with metastatic disease. Complete removal of the primary tumour and mesenteric metastases has been shown to increase survival in some studies^13^ ^14^, ^17^ ^22^, ^23^, ^24^. Whether hepatic cytoreductive surgery improves survival is controversial^6^ ^25^, ^26^, ^27^. Tumour debulking surgery has also been advocated as a worthwhile procedure in patients with severe carcinoid syndrome^5^ ^28^. The majority of these studies are observational and at risk of considerable bias, and, even though R0 resection is initially postulated, recurrence is common.

The aim in this case–control study was to assess prognostic factors presenting after resection of the primary tumour using population‐based data from the Swedish Cancer Registry (SCR).

## Methods

This was a case–control study with data retrieved from the SCR and the Swedish Cause of Death Registry (SCDR). The SCR is a nationwide registry, requiring clinicians and pathologists to report newly diagnosed tumours. It was created in 1958 and patients are indexed by means of their unique national registration number. The overall coverage of the registry has increased from 95·5 per cent in 1978^29^ to nearly 100 per cent in 2014^30^. The SCDR has existed in some form in Sweden since 1751. Between 1911 and 2003 the register was produced by Statistics Sweden, a government agency. Until 1991 a cause of death certificate was required before burial, making the registry essentially complete; following the legislative change in 1991 the registry has been about 99·1 per cent complete^31^.

Patients with date of diagnosis of SI‐NET between 1961 and 2001 in the SCR were eligible for inclusion in this study. Data on cause of death were extracted from the SCDR. All patients who were thought likely to have died from SI‐NETs were retrieved from the databases and included if they met the inclusion criteria. Inclusion criteria were: diagnosis of SI‐NET according to the SCR, and death resulting from the SI‐NET, confirmed after review of patient records. Exclusion criteria were: survival with SI‐NET for less than 1 month; cause of death other than SI‐NET more likely, or cause of death uncertain; no possibility of matching the patient with a control; missing or incomplete patient records; or did not have surgery.

Cases were defined as patients who had undergone surgery for SI‐NET and later died from the SI‐NET. Controls were patients with a diagnosed SI‐NET, matched to cases by calendar year of diagnosis and age at diagnosis, and outliving the matched case by at least 1 month. Sex was not used as a matching variable to avoid overmatching (matching of too many variables impedes comparison of these variables). Cases were matched to controls with a 1 : 1 ratio. Patient records were collected from the hospitals where the diagnosis had been made and, in many cases, from tertiary centres that had been involved in patient care. All records were reviewed by the principal author.

Ethical approval was obtained from the local ethics committee of the University of Uppsala (UPS02‐066).

### Study parameters

Studied parameters included macroscopically radical abdominal surgery, Symptom Severity Scale (SSS) score, European Neuroendocrine Tumor Society (ENETS) stage and 24‐h urinary 5‐hydroxyindoleacetic acid (5‐HIAA) concentration at 3 months, 2 and 5 years after surgery, and insufficiency of the tricuspid valve, evident on cardiac ultrasonography.

A macroscopically radical abdominal operation was defined as one where the operative report stated no remaining tumour or metastases in the abdomen, including mesenteric metastases, liver metastases, peritoneal carcinomatosis or other solid‐organ metastasis within the abdomen (R0).

A second malignancy, the finding of a second primary tumour, confirmed by biopsy or surgical specimen, was noted.

The main outcome of this study was the finding of prognostic factors of hormonally symptomatic disease and stage IV tumour.

Data collected from clinical records, laboratory protocols and radiology reports were recorded in Filemaker^®^ Pro (Filemaker, Santa Clara, California, USA).

The degree of symptomatic disease was evaluated using a modified Carcinoid SSS (*Table* 1), used in previous studies^11^ ^25^, ^32^ ^33^. Symptoms were scored at 3 months, 2 and 5 years after surgery. Not all patients were seen at these exact intervals, and therefore 3 months was defined as 1–6 months, 2 years as between 6 months and 3 years, and 5 years as between 3 and 5 years after surgery. If there were multiple entries about symptoms, the one closest to the respective time interval was chosen. When there was uncertainty regarding frequency of symptoms, the score correlating to lesser frequency was chosen. All scoring was done by the first author to eliminate inter‐reviewer discrepancies. Carcinoid heart disease was defined as insufficiency of the tricuspid valve, diagnosed by cardiac ultrasonography.

**Table 1:** Carcinoid Symptom Severity Scale (adapted from Schell et al.^32^ and Wessels and Schell^33^)

Score	Description	Symptoms	Frequency	Lifestyle effects
1	No symptoms	None	0	None
2	Mild symptoms	Diarrhoea, flushing or wheezing	1–4 times daily	None to minimal
3	Symptoms impact on daily living	Diarrhoea, flushing or wheezing	5–7 times daily	Restricts patient from leaving home for prolonged periods of time
4	Severe symptoms	Diarrhoea, flushing or wheezing	Multiple daily episodes (more than 7)	Symptoms require significant reorganization of daily activities to accommodate them; patient rarely leaves home, must be close to bathroom facilities and medical supplies
5	Disabling symptoms	Diarrhoea, flushing and wheezing	Multiple daily episodes	Symptoms are disabling; patient is unable to leave home or requires hospitalization

Patients were grouped into TNM stages, in accordance with current ENETS recommendations^34^. Initial staging of patients was generally difficult, and upstaging to stage IV was common at all follow‐up intervals.

Ki‐67, a marker of cell proliferation, was generally expressed as a Ki‐67 index. Tumours were consequently redetermined as grades according to ENETS guidelines^34^, which classify tumours as G1 (2 per cent or less), G2 (between 2 and 20 per cent) and G3 (more than 20 per cent) based on the Ki‐67 index, related to cell proliferation.

5‐HIAA levels in 24‐h collections of urine were measured in multiple laboratories during the study period, and their respective reference values differed over time. To construct comparable data, values were recalculated as either ‘normal’ or ‘raised’ in relation to the corresponding reference value.

5‐HIAA levels and symptoms were deemed to have the same causal pathway: symptomatic patients generally have higher 5‐HIAA values^25^. Therefore, 5‐HIAA was not included in the multivariable analysis.

Records of a different malignancy, documented after the diagnosis of a SI‐NET, were included in the database and analysed as a second malignancy.

### Statistical analysis

Data analyses were performed in RStudio^®^ version 0.99.893 (https://www.rstudio.com/) using conditional logistic regression to calculate odds ratios (ORs) with 95 per cent confidence intervals. A relationship was considered statistically significant when the c.i. did not include 1·00. In addition, multivariable analyses were calculated according to the SSS, ENETS stage, and sex. Some studied factors could have appeared at any time during postoperative follow‐up, and these were calculated for the whole postoperative period. Some data were missing from some patients, necessitating the need to deal with missing values. In this study, missing values were included in the analysis as a ‘missing value’ category. The analyses were focused on the situation in the postoperative phase, thereby excluding preoperative and perioperative findings. Baseline values have been described in a previous publication^11^. Data from different time periods were normalized by patient matching.

## Results

**Figure 1:**
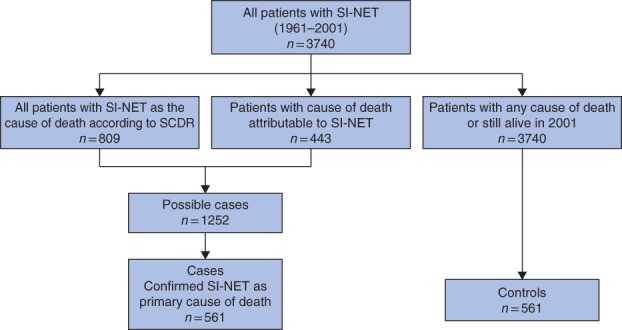
Flow chart of the matching process. SI‐NET, small intestinal neuroendocrine tumour; SCDR, Swedish Cause of Death Registry

Some 809 patients had SI‐NET listed as their most probable cause of death. After scrutinizing the causes of death for the remaining patients, a further 443 patients were identified whose death was likely to have been due to SI‐NET but had not been defined as such in the SCDR. As these patients had been diagnosed with SI‐NET during their lifetime, they were also included as cases unless they met the exclusion criteria. Finally, 561 cases were incorporated into the study together with 561 matched controls (*Fig*. 1).

All patients included in the study had surgical treatment, mostly involving resection of the primary tumour (842 patients). Mesenteric lymph node metastases were generally resected along with the primary tumour, and noted when resection was radical (579). Surgery for liver metastasis was rare at primary surgery^29^.

Data on symptoms, ENETS grade, 5‐HIAA level and sex were analysed at 3 months, 2 and 5 years after surgery. Only six patients had a known Ki‐67 value listed in the pathology report at the primary surgery. Therefore, ENETS grade was not included in univariable and multivariable analyses.

Of 877 patients who could be staged, 764 (87·1 per cent) had stage IIIB or IV disease. Including all stages in the analysis did not result in a significant risk reduction for stages I–IIIA compared with stages IIIB and IV (*Table* S1, supporting information). Therefore, patients were grouped as having either stage I–IIIB or stage IV disease. Patients who had died by the respective time intervals were excluded from further analysis, together with their corresponding controls (39 patients had died 3 months after surgery, 170 at 2 years and 311 at 5 years). General data on cases and controls are shown in *Table* 2.

**Table 2:** General data

	Cases (*n* = 561)	Controls (*n* = 561)
Age at diagnosis (years)*	66·8(10·5)	67·0(10·2)
Sex		
M	262 (46·7)	240 (42·8)
F	299 (53·3)	321 (57·2)
Length of survival (years)*, †	5·15(5·27)	11·0(6·9)
ENETS stage at presentation		
I	1 (0·2)	7 (1·2)
IIA	6 (1·1)	15 (2·7)
IIB	21 (3·7)	39 (7·0)
IIIA	6 (1·1)	18 (3·2)
IIIB	126 (22·5)	208 (37·1)
IV	291 (51·9)	139 (24·8)
Insufficient data to stage	110 (19·6)	135 (24·1)
SSS score‡		
1	311 (55·4)	386 (68·8)
2	135 (24·1)	76 (13·5)
3	54 (9·6)	26 (4·6)
4	24 (4·3)	7 (1·2)
Missing data	37 (6·6)	66 (11·8)

Values in parentheses are percentages unless indicated otherwise;

* values are mean(s.d.).

† Some control patients were alive at study termination and have been excluded.

‡ Modified Symptom Severity Scale (SSS), according to Schell *et al*.^32^ and Wessels and Schell^33^.

ENETS, European Neuroendocrine Tumor Society.

### Carcinoid heart disease

Tricuspid valve insufficiency was recorded in 48 cases (8·6 per cent) *versus* 27 controls (4·8 per cent). Carcinoid heart disease was more common in cases, with an OR of 1·91 (*Table* 3). These data were not included in multivariable analyses because cardiac ultrasonography was performed at random time intervals (if at all), and at the discretion of the treating physicians.

**Table 3:** Patient data for the whole postoperative period

	Cases (*n* = 561)*	Controls (n = 561)*	Univariable odds ratio†
Sex			
M	262 (46·7)	240 (42·8)	1·00 (reference)
F	299 (53·3)	321 (57·2)	0·86 (0·68, 1·08)
Confirmed insufficiency oftricuspid valve
Yes	48 (8·6)	27 (4·8)	1·91 (1·16, 3·17)
No	513 (91·4)	534 (95·2)	1·00 (reference)
Second primary			
Yes	21 (3·7)	56 (10·0)	0·37 (0·22, 0·61)
No	432 (77·0)	406 (72·4)	1·00 (reference)
Missing data	108 (19·3)	99 (17·6)	1·05 (0·77, 1·44)
Macroscopically radical aftersecondary surgery‡	(*n* = 73)	(*n* = 73)	
Yes	12 (16)	28 (38)	1·00 (reference)
No	48 (66)	32 (44)	3·28 (1·42, 7·56)
Missing data	13 (18)	13 (18)	2·12 (0·67, 6·72)

Values in parentheses are *percentages and †95 per cent confidence intervals.

‡ Includes only patients who had secondary or follow‐up surgery.

### Secondary surgery

If the patient had secondary surgery, regardless of radicality at the primary operation, there was an increased risk of death in the group that did not have macroscopically radical excision (OR 3·28). This was a subanalysis of only matched pairs of cases and controls, where both had a secondary operation, and was not included in the multivariable analysis.

### Second malignancy

Overall, 77 (6·9 per cent) of the 1122 patients developed a second malignancy during the study period. As this was more common among the controls (10·0 per cent *versus* 3·7 per cent in cases), a second primary was a prognostic factor for not dying from SI‐NET (OR 0·37) (*Table* 3).

### Prognostic factors at 3 months

Hormone‐related symptoms still present 3 months after surgery were a significant prognostic factor for death. There was a trend towards a stronger prognostic effect for increasing SSS score (*Table* 4).

**Table 4:** Patient data at 3 months after surgery

	Cases (*n* = 522)*	Controls (*n* = 522)*	Odds ratio†	
			Univariable	Multivariable
Sex				
F	282 (54·0)	297 (56·9)	1·00 (reference)	1·00 (reference)
M	240 (46·0)	225 (43·1)	1·12 (0·89, 1·42)	1·11 (0·86, 1·44)
SSS score‡				
1	211 (40·4)	295 (56·5)	1·00 (reference)	1·00 (reference)
2	123 (23·6)	74 (14·2)	2·39 (1·68, 3·39)	1·71 (1·17, 2·50)
3	29 (5·6)	15 (2·9)	3·10 (1·54, 6·27)	2·09 (1·00, 4·39)
4–5§	9 (1·7)	2 (0·4)	5·54 (1·18, 25·99)	3·82 (0·69, 21·04)
Missing data	150 (28·7)	136 (26·1)	1·68 (1·25, 2·26)	1·78 (1·25, 2·52)
ENETS stage				
I–IIIB	147 (28·2)	262 (50·2)	1·00 (reference)	1·00 (reference)
IV	270 (51·7)	133 (25·5)	3·92 (2·85, 5·41)	3·44 (2·46, 4·80)
Missing data	105 (20·1)	127 (24·3)	1·52 (1·09, 2·13)	1·20 (0·82, 1·76)
5‐HIAA level				
Normal	88 (16·9)	141 (27·0)	1·00 (reference)	Not included
Raised	107 (20·5)	60 (11·5)	2·90 (1·92, 4·40)	
Missing data	327 (62·6)	321 (61·5)	1·70 (1·25, 2·31)	

Values in parentheses are *percentages and †95 per cent confidence intervals.

‡ Adapted Symptom Severity Scale (SSS), as described by Schell *et al*.^32^ and Wessels and Schell^33^.

§ SSS scores 4 and 5 were grouped together because of the scarcity of patients with severe symptoms. Variables included in the multivariable analysis were sex, SSS score, European Neuroendocrine Tumor Society (ENETS) stage and age; 5‐hydroxyindoleacetic acid (5‐HIAA) was not included in the multivariable analysis as it probably shares a causal pathway with symptoms.

With regard to ENETS staging, stage IV disease carried a significantly increased risk of death with an OR of 3·92 (95 per cent c.i. 2·85 to 5·41) at 3 months. This finding remained statistically significant in multivariable analysis (*Table* 4).

A postoperative increase in 5‐HIAA concentration was associated with a significantly higher risk of death (OR 2·90) in univariable analysis, indicating a potential prognostic impact for this factor.

### Prognostic factors at 2 years

At 2 years, the presence of symptoms and stage IV disease remained prognostic factors, although not quite significant for SSS score 3 in multivariable analysis (OR 2·19, 95 per cent c.i. 0·97 to 4·93). The trend of increasing ORs for death with more symptomatic disease remained, and patients with the most severe symptoms at 2 years had a univariable OR of 13·88 (OR 11·387 in multivariable analysis) compared with their asymptomatic peers. In univariable analysis, raised 5‐HIAA values were more common in patients who died from SI‐NET (OR 2·55) (*Table* 5).

**Table 5:** Patient data at 2 years after surgery

	Cases (*n* = 394)*	Controls (*n* = 394)*	Odds ratio†	
			Univariable	Multivariable
Sex				
F	225 (57·1)	235 (59·6)	1·00 (reference)	1·00 (reference)
M	169 (42·9)	159 (40·4)	1·11 (0·85, 1·46)	1·16 (0·86, 1·56)
SSS score‡				
1	119 (30·2)	185 (47·0)	1·00 (reference)	1·00 (reference)
2	95 (24·1)	66 (16·8)	2·36 (1·55, 3·62)	1·73 (1·10, 2·73)
3	22 (5·6)	12 (3·0)	3·24 (1·49, 7·06)	2·19 (0·97, 4·93)
4–5§	11 (2·8)	1 (0·3)	13·88 (1·77, 108·88)	11·38 (1·41, 91·93)
Missing data	147 (37·3)	130 (33·0)	1·81 (1·28, 2·57)	1·78 (1·22, 2·60)
ENETS stage				
I–IIIB	126 (32·0)	193 (49·0)	1·00 (reference)	1·00 (reference)
IV	179 (45·4)	100 (25·4)	3·01 (2·09, 4·33)	2·54 (1·72, 3·75)
Missing data	89 (22·6)	101 (25·6)	1·45 (1·00, 2·11)	1·19 (0·79, 1·80)
5‐HIAA level				
Normal	56 (14·2)	95 (24·1)	1·00 (reference)	Not included
Raised	82 (20·8)	55 (14·0)	2·55 (1·57, 4·15)	
Missing data	256 (65·0)	244 (61·9)	1·86 (1·27, 2·74)	

Values in parentheses are *percentages and †95 per cent confidence intervals.

‡ Adapted Symptom Severity Scale (SSS), as described by Schell *et al*.^32^ and Wessels and Schell^33^. Variables included in the multivariable analysis were: sex, SSS score, European Neuroendocrine Tumor Society (ENETS) stage and age; 5‐hydroxyindoleacetic acid (5‐HIAA) was not included in the multivariable analysis as it probably shares a causal pathway with symptoms.

§ SSS scores 4 and 5 were grouped together because of the scarcity of patients with severe symptoms.

### Prognostic factors at 5 years

At 5 years, symptomatic disease and ENETS stage were still important prognostic factors for death. Increased 5‐HIAA values remained indicative of death from SI‐NET (*Table* 6).

**Table 6:** Patient data at 5 years after surgery

	Cases (*n* = 264)*	Controls (*n* = 264)*	Odds ratio†	
			Univariable	Multivariable
Sex				
F	152 (57·6)	158 (59·8)	1·00 (reference)	1·00 (reference)
M	112 (42·4)	106 (40·2)	1·04 (0·75, 1·44)	1·16 (0·81, 1·66)
SSS score‡				
1	55 (20·8)	116 (43·9)	1·00 (reference)	1·00 (reference)
2	52 (19·7)	42 (15·9)	2·61 (1·51, 4·53)	2·58 (1·45, 4·59)
3	26 (9·8)	11 (4·2)	5·52 (2·38, 12·82)	3·63 (1·51, 8·76)
4–5§	6 (2·3)	1 (0·4)	10·15 (1·16, 88·38)	5·40 (0·63, 46·56)
Missing data	125 (47·3)	94 (35·6)	2·83 (1·84, 4·34)	2·93 (1·83, 4·70)
ENETS stage				
I–IIIB	83 (31·4)	130 (49·2)	1·00 (reference)	1·00 (reference)
IV	113 (42·8)	65 (24·6)	2·94 (1·75, 4·94)	2·94 (1·75, 4·94)
Missing data	68 (25·8)	69 (26·1)	1·73 (1·10, 2·72)	1·28 (0·77, 2·11)
5‐HIAA level				
Normal	33 (12·5)	56 (21·2)	1·00 (reference)	Not included
Raised	60 (22·7)	27 (10·2)	3·53 (1·86, 6·71)	
Missing data	171 (64·8)	181 (68·6)	1·60 (0·99, 2·58)	

Values in parentheses are *percentages and †95 per cent confidence intervals.

‡ Adapted Symptom Severity Scale (SSS), as described by Schell *et al*.^32^ and Wessels and Schell^33^. Variables included in the multivariable analysis were: sex, SSS score, European Neuroendocrine Tumor Society (ENETS) stage and age; 5‐hydroxyindoleacetic acid (5‐HIAA) was not included in the multivariable analysis as it probably shares a causal pathway with symptoms.

§ SSS scores 4 and 5 were grouped together because of the scarcity of patients with severe symptoms.

## Discussion

Symptomatic disease and stage IV disease were confirmed as prognostic factors for death in patients with SI‐NET for at least 5 years after surgery. All symptom scoring was done by the principal author because retrospective scoring of symptoms based on medical charts might be investigator‐dependent. Scoring symptoms in this way should make for a more balanced estimation of symptom severity.

Symptomatic disease was a prognostic factor in patients with SI‐NETs even after adjusting for tumour stage, suggesting that hormonally active disease following resection presents a threat to the patient. This finding raises the question of whether tumour‐debulking surgery in patients with symptomatic and disseminated disease might have an additional survival benefit besides alleviating symptoms. It was shown in a previous study^11^ that preoperative symptoms were a negative prognostic factor. This emphasizes the importance of treating symptoms. Those with the most severe symptoms were most likely to die from their SI‐NET. The confidence intervals were wide in multivariable analysis because there were few records of severe symptoms. Data on 5‐HIAA values were missing in over 60 per cent of the patients at all time intervals; this would complicate the analysis, and 5‐HIAA was not included in the multivariable analysis as there is probably a common causal pathway for symptoms and circulating serotonin levels.

Tricuspid valve insufficiency has been shown^13^ to increase with increasing symptomatology, and in the present study also represented a potential prognostic factor for death from SI‐NET, although not verified by multivariable analysis. The number of patients with tricuspid valve insufficiency was relatively low, probably a result of the long study interval and because cardiac ultrasonography was not performed routinely in the early part of the study.

Over time, and if surveillance is long enough, many patients develop recurrence^5^. Consistent follow‐up is important, as a second macroscopically radical resection appeared to improve survival. The importance of macroscopically radical surgery for SI‐NETs has been emphasized previously^18^ ^26^. In the present study, there seemed to be a survival benefit of follow‐up surgery if the intent was to achieve a tumour‐free abdomen. This must be interpreted with caution. A nested case–control study is not designed to study therapeutic effects, and both the types of surgery and their indications were heterogeneous in this study, limiting the ability to draw robust conclusions.

It is striking that such a large proportion of the patients (30·3 per cent) died within 2 years of their first operation. ENETS stage and grade system alone seem insufficient to predict survival. There will be a substantial difference in survival within stages. Adding symptomatic disease to the ENETS stage and grade system might increase its prognostic accuracy.

In the present study, the finding of increased survival in patients with a second malignancy was surprising. It is not the authors' belief that harnessing two malignant tumours infers a survival benefit. This finding is more likely due to the design of the study rather than an actual biological effect. As the study required that the patients (cases) had died from the SI‐NET, controls were more likely to have died from other causes, such as another malignancy. Death from a more malignant tumour could therefore only occur among the controls, who were accepted as controls only if they outlived their corresponding cases. This resulted in the observed survival benefit of having a second malignancy.

This nested case–control study, based on the national SCR, eliminates some bias inherent to most retrospective studies, but still has important limitations. One important issue is missing values. There are three general methods of handling cases and controls not having complete data for all variables. The method used in this study was to include missing values as an additional category. Other methods are either to exclude missing variables, a complete case analysis, or to impute data at random, adjusted for known variables that might affect the unknown variable. All three methods have their limitations and infer the risk of bias. The problem with the additional variable analysis used in the present study is that it infers the risk of the method underestimating the true variance in the data, thereby estimating false probabilities. For this reason, variables included in multivariable analysis were denoted prognostic factors only if they remained significant in multivariable analysis. The complete case analysis has the weakness of including a potential selection bias into the analysis and might overestimate or underestimate the effect of the studied variable when the numbers of missing data were unequal between cases and controls. Finally, for weighted, randomly computed, missing data, the computational exercise of calculating variables might produce false estimates of probability, depending on which variables were included in the weighting exercise and how they were weighted against one another, whilst possibly underestimating variance, and producing a type I error.

## Supporting information


**Table S1** Univariable analysis of all disease stages
