# Supplementary material for: Prognostic factors for death after surgery for small intestinal neuroendocrine tumours
Source: BJS Open. 2018 May 28;2(5):345–52. doi: 10.1002/bjs5.76 (PMC6156160; doi:10.1002/bjs5.76)
Supplement: Supplementary file 1 — Table S1 Univariable analysis of all disease stages [file BJS5-2-345-s001.docx]

**BJS5_76**

**Prognostic factors for death after surgery for small intestinal neuroendocrine tumours**

**J. Eriksson, J. E. H. Garmo, C. Ihre-Lundgren and P. Hellman**

**Table S1** Univariable analysis of all disease stages

|  | Cases | | Controls | | Total | | Odds Ratio Univariable (OR UV) | |
| --- | --- | --- | --- | --- | --- | --- | --- | --- |
| Patient data | n | % | n | % | n | % | OR UV | CI |
| ENETS stage at three months | | | | | | | | |
| Stage I-IIIA | 29 | 5.6 | 72 | 13.8 | 101 | 9.5 | 1.00 | Reference |
| Stage IIIB | 118 | 22.6 | 190 | 36.4 | 308 | 29.5 | 1.60 | 0.94 - 2.71 |
| Stage IV | 270 | 51.7 | 133 | 25.5 | 403 | 38.6 | 5.53 | 3.26 - 9.37 |
| Insufficient to stage | 105 | 20.1 | 127 | 24.3 | 232 | 22.2 | 2.13 | 1.25 - 3.64 |
| ENETS stage at two years | | | | | | | | |
| Stage I-IIIA | 24 | 6.1 | 53 | 13.5 | 77 | 9.8 | 1.00 | Reference |
| Stage IIIB | 102 | 25.3 | 140 | 35.5 | 242 | 30.7 | 1.64 | 0.91 - 2.95 |
| Stage IV | 179 | 25.9 | 100 | 25.4 | 279 | 35.4 | 4.34 | 2.41 - 7.79 |
| Insufficient to stage | 89 | 45.4 | 101 | 25.6 | 190 | 24.1 | 1.98 | 1.10 - 3.58 |
| ENETS stage at five years | | | | | | | | |
| Stage I-IIIA | 15 | 5.7 | 40 | 15.2 | 55 | 10.2 | 1.00 | Reference |
| Stage IIIB | 83 | 31.4 | 130 | 49.2 | 213 | 40.3 | 2.27 | 1.07 - 4.82 |
| Stage IV | 114 | 42.2 | 65 | 24.1 | 179 | 33.1 | 6.03 | 2.78 - 13.12 |
| Insufficient to stage | 68 | 25.8 | 69 | 26.1 | 137 | 25.9 | 3.06 | 1.40 - 6.66 |
| CI: 95% Confidence Interval.  ENETS, European Neuroendocrine Tumor Society. | | | | | | | | |
